# The Antimicrobial Efficacy of Amine-Containing Surfactants Against Cysts and Trophozoites of *Acanthamoeba* spp.

**DOI:** 10.3390/microorganisms13030665

**Published:** 2025-03-15

**Authors:** Dharanga Ratnayake, Michael Ansah, Brian Batham, Daniel Keddie, Gavin McNee, Wayne Heaselgrave

**Affiliations:** 1 Department Biomedical Science, University of Wolverhampton, Wulfruna Street, Wolverhampton WV1 1LY, UK; d.r.ratnayake@wlv.ac.uk (D.R.); m.l.ansah@wlv.ac.uk (M.A.); b.j.batham@wlv.ac.uk (B.B.); 2 School of Chemistry, University of Nottingham, University Park, Nottingham NG7 2RD, UK; daniel.keddie@nottingham.ac.uk; 3 Department Biomedical Sciences, School of Infection, Inflammation & Immunology, College of Medicine & Health, University of Birmingham, Edgbaston, Birmingham B15 2TT, UK

**Keywords:** *Acanthamoeba*, contact lens, keratitis

## Abstract

Microbial keratitis, a vision-threatening infection commonly linked to contact lens use, poses a significant challenge, particularly when caused by *Acanthamoeba* species. Acanthamoeba keratitis (AK) is difficult to treat due to the organism’s ability to form resilient cysts, necessitating prolonged and complex therapeutic interventions. This study evaluated novel amidopropyl dimethylamines (APDs) and amidopropyl quaternary trimethylammoniums (APTs) for their antimicrobial efficacy against *Acanthamoeba castellanii* and *Acanthamoeba polyphaga* cysts. Minimum effective concentrations were determined, and time–kill assays assessed microbial inactivation over 24 h. The results indicated that certain APTs, particularly elaidamidopropyl trimethylammonium (EAPT) and oleamidopropyl trimethylammonium (OAPT), demonstrated superior cysticidal activity compared to the commercially used MAPD, achieving greater log reductions within 24 h (*p* < 0.0001) at a concentration of 25 µM. The enhanced efficacy of these compounds is potentially attributed to their unsaturated alkyl chains and positive charge, improving antimicrobial activity through the greater disruption of the *Acanthamoeba* cell membrane. These findings highlight the potential of APTs as alternative agents for incorporation into multipurpose lens disinfectants and AK treatment, offering improved disinfection efficacy. Further investigation is justified to optimise formulations for clinical and commercial applications.

## 1. Introduction

Global contact lens use is rising, with approximately 4.5 million wearers in the UK and over 41 million in the USA [1,2]. This increase correlates with a higher incidence of eye infections such as microbial keratitis, which affects the cornea and is estimated at 1.5 to 2 million cases globally per year [3]. Contact lens wearers are particularly susceptible to microbial keratitis due to risks associated with improper handling and poor lens hygiene [4,5]. Pathogens causing keratitis include Gram-positive bacteria such as *Staphylococcus aureus*, Gram-negative bacteria like *Pseudomonas aeruginosa*, fungal species including *Candida albicans* and *Fusarium keratoplasticum*, and free-living amoebae, notably *Acanthamoeba* spp. [6,7].

Acanthamoeba keratitis (AK) is a severe, vision-threatening infection predominantly affecting contact lens users, who account for 90% of cases [8]. In the USA, the AK incidence rate is around two cases per million lens wearers annually [9]. A recent study in East England recorded an annual microbial keratitis incidence of 6.96 per 100,000 population/year, with *Acanthamoeba* responsible for 1.1% of these cases [10]. Treatment for microbial keratitis including AK is challenging due to the resistance of *Acanthamoeba* cysts, often requiring over five months of treatment; about 15% of cases need surgical intervention, with a significant vision loss in 30% [11,12]. Given these challenges, the need for improved therapeutic strategies is evident, particularly for agents effective against cyst stages.

### 1.1. Amidopropyl Dimethylamine Compounds

Amine-containing surfactants, specifically tertiary amines and related compounds, represent a promising area of research [13]. These compounds, derived from natural plant and animal fatty acids, are widely used in personal hygiene products for their conditioning and antimicrobial properties. Structurally, these surfactants consist of a fatty acid-derived carbon chain, with variable head groups influencing chemical properties and functionality [14]. For instance, amidoamines like myristamidopropyl dimethylamine (MAPD) show activity against a range of pathogens, including *P. aeruginosa*, *S. aureus*, *C. albicans,* and *Acanthamoeba* spp. [15,16].

### 1.2. Quaternary Ammonium Compounds

Quaternary ammonium compounds (QACs), a well-established class of cationic surfactants, act similarly by adsorbing to microbial cell walls, disrupting membrane integrity, and causing cytoplasmic leakage [17]. This mechanism results in the breakdown of essential proteins and nucleic acids, leading to cell lysis and death [18]. Over successive generations, QACs have been refined for greater potency and broader application, notably in hard-surface disinfection and multipurpose solutions [18,19]. Both QACs and amidoamines are advantageous due to their non-specific, multi-targeted actions, which reduce the likelihood of resistance development [19].

Research into amidoamines, particularly fatty acid amidopropyl derivatives, has led to the synthesis of amidoamine compounds as novel anti-microbial agents in our laboratory. These amidoamines demonstrate similar cationic surfactant properties, with the potential ability to target and disrupt microbial membranes, making them promising candidates for treating Acanthamoeba keratitis (AK) and other bacterial/fungal pathogens causing microbial keratitis [13]. Their potential broad-spectrum antimicrobial activity, followed by their likely inclusion in multipurpose disinfectant solutions and topical treatments for contact lens users, offers a significant opportunity to improve infection prevention, thereby reducing the risk of microbial keratitis among wearers [15,20].

In this study, we investigated the structure–activity relationship between fatty acid amidopropyl dimethylamines by synthesising and testing compounds of varying fatty acid chain length and saturation against *Acanthamoeba* and other ocular pathogens. Additional studies comparing fatty acid amidopropyl tertiary amines with their respective quaternary trimethylammoniums were also performed. The difference between the tertiary and quaternary derivatives is that whilst tertiary amine derivatives are charged once in solution through protonation, the quaternary derivatives are permanently charged with the addition of a methyl group. This ongoing research into amidoamine synthesis continues to explore the potential of these compounds as therapeutic agents for treating and preventing microbial keratitis.

## 2. Materials and Methods

### 2.1. Test Organism Strains and Culture

The *Acanthamoeba* strains *A. polyphaga* (ATCC 30461) and *A. castellanii* (ATCC 50370) were sourced from the American Type Culture Collection (LGC Standards, Teddington, UK). Trophozoites were cultured in tissue culture flasks with Ac#6 medium at 30 °C in a static incubator. To induce cyst formation, cells were transferred to Neff’s encystment medium (NEM) in tissue culture flasks and incubated at 30 °C in a shaking incubator, following a previously published method [13].

### 2.2. Synthesis Reagents

The reagents used to synthesise the APDs and APTs in this study were obtained from the following manufacturers: DMAPA, toluene, hexane, stearic acid, myristic acid and palmitic acid (Sigma-Aldrich, Gillingham, UK). Elaidic acid, oleic acid and linolenic acid (VWR Ltd., Leicestershire, UK). Lauric acid, decanoic acid, linoleic acid, didodecyldimethylammonium bromide, dimethyldioctadecylammonium bromide, benzyldimethylhexadecylammonium chloride hydrate, decyltrimethylammonium bromide, dodecyltrimethylammonium chloride, (1-tetradecyl) trimethylammonium bromide and (1-hexadecyl) trimethylammonium chloride (Thermo Fisher Scientific, Altrincham, UK).

### 2.3. Amidopropyl Dimethylamine Synthesis

The synthesis of amidopropyl dimethylamines was performed following the methodology provided by Muzyczko, Shore and Loboda (1968) [21]. In brief, solutions of dimethylaminopropylamine (DMAPA) and fatty acid (at a stoichiometry of 1.4:1.0 moles, respectively) were refluxed in toluene for 3 h using the Dean–Stark apparatus. Toluene and unreacted DMAPA were removed by rotary evaporation at 65 °C. The amidopropyl dimethylamines were then dried in a vacuum oven to a constant weight. The products were then characterised using hydrogen and carbon NMR.

### 2.4. Quaternary Ammonium Synthesis

Quaternary ammonium compounds feature a central nitrogen atom bonded to four distinct moieties. In this case, amidopropyl quaternary ammoniums (APTs) are synthesised by adding a methyl group (CH_3_) to the terminal nitrogen of amidopropyl dimethylamine (APD). The synthesis of APTs involves a reaction between APD and iodomethane. Four APDs—MAPD, PAPD, EAPD, and OAPD—were converted to APTs to evaluate whether the conversion to a permanently cationic form enhances antimicrobial activity.

### 2.5. Minimum Trophozoite Amoebicidal Concentration (MTAC) Assay

The test compounds were added to a 96-well microtitre plate (Helena Biosciences, Gateshead, UK) and a series of two-fold serial dilutions of each of the test compounds were performed using ¼ strength Ringer’s solution (Oxoid, Basingstoke, UK). After adjusting the trophozoite cell count to a concentration of 2 × 10^4^ cells/mL, the trophozoites were then re-suspended in an Ac#6 growth medium and 100 µL of cells were then added to all the wells in the plate, resulting in a final trophozoite concentration of 1 × 10^4^ cells/mL. After adding the cells, the final drug concentrations ranged from 500 μg/mL to 0.49 μg/mL. The plate was then incubated at 32 °C for 24 h. The MTAC was then determined by comparing the rate of growth, inhibition, or kill against the control, using an inverted microscope (×200). The MTAC was defined as the lowest concentration that caused cell lysis relative to the control. This is easily seen microscopically, as healthy trophozoites remain attached to the bottom of the wells. The control wells received a diluent in the absence of a test compound, and any differences relative to the control can be confirmed microscopically through the absence of cell division and the presence of cell lysis.

### 2.6. Minimum Cysticidal Concentration (MCC) Assay

After adjusting the cyst count to a concentration of 2 × 10^4^ cells/mL in ¼ strength Ringer’s solution, 100 µL of cysts was then added to the wells, resulting in a final cyst concentration of 1 × 10^4^ cells/mL. The control wells received a diluent in the absence of a test compound. After adding the cysts, the plates were incubated at 32 °C for 48 h. After 48 h of incubation, the liquid was aspirated from the microtitre plate using a Vacusip (Integra Biosciences, Thatcham, UK), leaving behind the cysts that adhered to the bottom of the wells. Wells were then refilled with ¼ strength Ringer’s solution, which was allowed to stand for 10 min before being aspirated. This step was repeated 3 times to ensure the removal of the test compounds. Finally, to each well, an *Escherichia coli* suspension at an OD of 0.1–0.2 at 600 nm was added. The plates were then incubated at 32 °C and observed daily for 7–14 days. The MCC was defined as the lowest concentration that resulted in no trophozoite excystation relative to the control, and any differences relative to the control was confirmed microscopically through the absence of excystation and cell division.

### 2.7. Acanthamoeba Time–Kill Assay

The rate of antimicrobial activity for each compound was investigated using the time–kill method as previously described [13]. In brief, this involved exposing *Acanthamoeba* cysts to a concentration of the test compound in Ringer’s solution for up to 24 h, and the control was diluent without test compounds. At time intervals of 0, 2, 4, 6, and 24 h, 20 μL aliquots were removed from each centrifuge tube in quadruplet, and serial 10-fold dilutions were performed across the plate. Each well was then seeded with *E. coli* and the plates were incubated at 32 °C for 7–14 days. After 14 days, the plates were examined for encystment or complete kill using an inverted microscopy. The number of viable organisms at each time point was calculated using the Spearman–Karber most probable number method [22].

### 2.8. Hep-2 Cell Cytotoxicity

The cytotoxic effects of the test compounds were evaluated using the Hep-2 human epithelial cell line (ECACC number 86030501), which was obtained from the European Collection of Cell Cultures (Centre for Applied Microbiology and Research, Salisbury, UK). The cells were cultured and maintained at 37 °C in minimum essential medium, supplemented with 10% heat-inactivated foetal bovine serum (Life Technologies Ltd., Paisley, Scotland). Confluent monolayers grown in flasks were used to seed a 96-well microtiter plate at a density of 1 × 10^4^ cells per well in 100 μL of growth medium, followed by incubation at 37 °C. After reaching approximately 75% confluence in the wells, the medium was replaced, and the cells were prepared for cytotoxicity testing. Serial two-fold dilutions of the test compounds in an appropriate solvent were added to the wells, and the plate was incubated at 37 °C for 24 h. Cytotoxicity was assessed using the CellTiter 96 AQueous One Solution Cell Proliferation Assay (Promega, Southampton, UK).

### 2.9. Analysis of Data

To determine the reduction in viable *Acanthamoeba* cysts, the decrease in viability was plotted as log viability for each of the time points, and the reduction was determined relative to the log viability. Statistical analysis was performed using GraphPad Prism Version 10 (La Jolla, CA, USA). The log reduction in the compounds was compared to the control and to each other and then analysed using the Tukey–Kramer multiple comparisons test.

## 3. Results

In this study, the synthesised amidoamine compounds were evaluated for their activity against trophozoites and cysts of *A. castellanii* (ATCC 50370), *A. polyphaga* (ATCC 30461) as well as for their toxicity against a Hep2 cell line. The test compounds were initially screened to identify the minimum concentration at which they exhibited activity against trophozoites and cysts. Following this, their inactivation kinetics were assessed over a 24 h period using a time–kill study.

### 3.1. Minimum Trophozoite Amoebicidal Concentration (MTAC) and Minimum Cysticidal Concentration (MCC)

The results in Table 1 display the antimicrobial activity of the synthesised tertiary amidoamines. The results were compared to that of Myristamidopropyl dimethylamine (MAPD) as this is currently used as the active ingredient in a number of commercially available contact lens solutions.

Myristamidopropyl dimethylamine (C14) required a concentration of 0.025 mM to show MTAC activity against both strains of trophozoites. Reduced activity was observed with the shorter chained Capramidopropyl dimethylamine (C10) and Lauramidopropyl dimethylamine (C12), with both requiring 0.244 mM and 0.055 mM, respectively.

All the unsaturated compounds (C18:1, C18:2, and C18:3) exhibited superior activity compared to their saturated counterpart SAPD (C18) against *Acanthamoeba* trophozoites and cysts. The lowest MTAC was recorded against Linolenamidopropyl dimethylamine (C18:3), Elaidamidopropyl dimethylamine (C18:1 *trans*) and Oleamidopropyl dimethylamine (C18:1 *cis*), where just 0.011 mM was required to demonstrate antimicrobial activity against trophozoites. However, *A. polyphaga* trophozoites showed reduced susceptibility to OAPD, requiring a slightly higher MTAC of 0.043 mM. LiAPD demonstrated superior activity against *A. polyphaga* trophozoites at 0.005 mM, which was also the lowest MTAC of all the APDs.

MAPD (C14) demonstrated the lowest MCC across both strains, with values of 0.050 and 0.100 mM for *A. castellanii* and *A. polyphaga* respectively. SAPD (C18) was the least effective compound, with the highest MCC against both *A. castellanii* (0.678 mM) and *A. polyphaga* (1.356 mM). EAPD (C18:1 *trans*), OAPD (C18:1 *cis*), LAPD (C18:2) and LiAPD (C18:3) showed reduced activity in the cyst screening assay relative to MAPD, with MCCs of 0.085, 0.085, 0.686 and 0.086 mM, respectively.

In the cytotoxicity assay, MAPD exhibited cytotoxicity at a concentration of 0.200 mM. In comparison, LAPD (C18:2) demonstrated the highest cytotoxicity at 0.343 mM. Among the APD compounds, EAPD and OAPD showed cytotoxicity levels of 0.021 mM. The lowest cytotoxicity was observed for SAPD at 0.011 mM.

Based on the cysticidal activity, MAPD, EAPD, and OAPD were then selected to be converted into the corresponding amidopropyl trimethylammoniums (Table 2) to observe if this would enhance antimicrobial activity.

MAPT required higher MTACs across both strains with 0.095 and 0.191 mM against *A. castellanii* and *A. polyphaga* trophozoites, respectively. OAPT and EAPT demonstrated superior activity against *Acanthamoeba* trophozoites with an MTAC of 0.003 mM and 0.005 mM, respectively. Against cysts, OAPT and EAPT displayed an MCC of 0.005–0.010 and 0.020 mM, respectively, against both species of cyst. With the cytotoxicity assay, all three compounds showed toxicity in the range 0.041–0.048 mM.

### 3.2. Time–Kill Assays

Only cysts were selected for the time–kill assays as they are considered the most resistant form of *Acanthamoeba.* Table 3 shows that at a concentration of 0.025 mM, all APDs exhibited a time-dependent reduction in *A. castellanii* viability over 24 h. However, MAPD, OAPD, and EAPD demonstrated the highest amoebicidal activity, with a 1.58–2.58 log_10_ reduction at 6 h and a 1.67–2.58 log_10_ reduction at 24 h. OAPD exhibited the greatest reduction, achieving a 2.58 log_10_ decrease at 24 h. In contrast, PAPD, CAPD, and SAPD demonstrated minimal reductions, with log_10_ values ranging from −0.08 to −0.75 at 24 h, suggesting lower amoebicidal activity. LaAPD and LnAPD showed moderate activity, with 1.33 and 1.42 log_10_ reductions, respectively, at 24 h.

Table 4 demonstrates EAPD and OAPD exhibiting the highest reductions in viability, with log_10_ reductions of 1.42 and 1.00 at 6 h and 2.50 and 2.17 at 24 h, respectively. MAPD also showed significant activity, with a 1.67 log_10_ reduction at 24 h. In contrast, CAPD, SAPD, and LnAPD displayed minimal reductions, with final log_10_ reductions ranging from 0.58 to 1.00, suggesting lower activity. PAPD, LAPD, and LaAPD exhibited moderate activity, with reductions between 1.17 and 1.67 log_10_ reductions at 24 h.

Table 5 shows all APTs demonstrating amoebicidal activity against *A. castellanii*, with increasing reductions in viability over 24 h. OAPT exhibited the highest activity, with a 1.58 log_10_ reduction at 6 h and a 3.25 log_10_ reduction at 24 h, indicating strong amoebicidal effects. EAPT followed closely, with a 1.42 log_10_ reduction at 6 h and a 2.92 log_10_ reduction at 24 h. MAPT demonstrated the lowest activity among the three, with a 1.08 log_10_ reduction at 6 h and a 2.58 log_10_ reduction at 24 h. These results suggest that OAPT is the most effective compound against *A. castellanii*.

Table 6 details the amoebicidal activity against *A. polyphaga* from all APTs, with increasing reductions in viability over 24 h. However, OAPT demonstrated the highest efficacy, with a 1.50 log_10_ reduction at 6 h and a 3.17 log_10_ reduction at 24 h. EAPT followed closely, showing a 1.33 log_10_ reduction at 6 h and a 2.92 log_10_ reduction at 24 h. MAPT displayed the lowest amoebicidal activity comparative to other APTs, with a 1.00 log_10_ reduction at 6 h and a 2.67 log_10_ reduction at 24 h. These results suggest that OAPT is the most potent APT against *A. polyphaga* under the test conditions.

To enable a clear comparison of the most active compounds (MAPD, OPAD, EAPD, MAPT, OAPT and EAPT), graphs were constructed to show the log reduction in viability over time.

**Figure 1:**
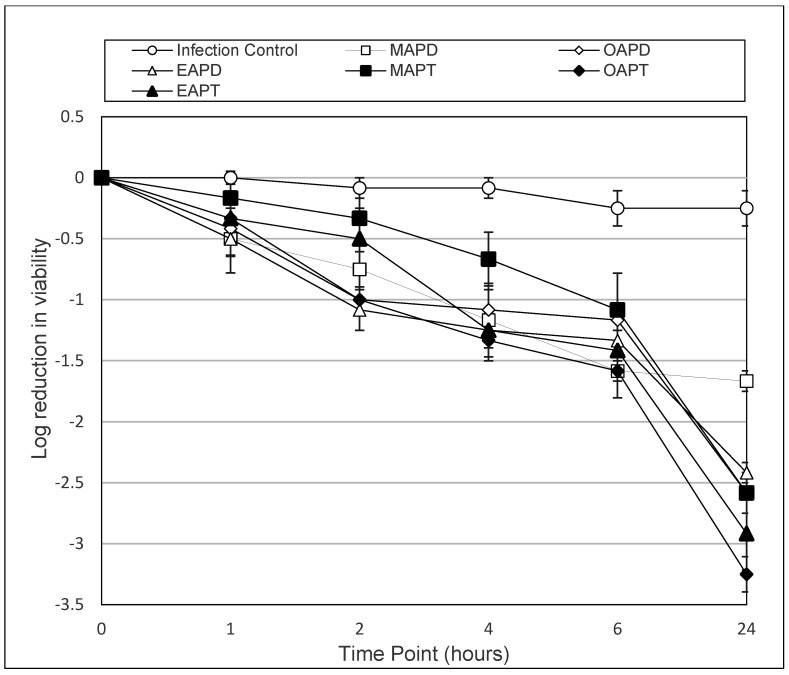
The above graph shows the antimicrobial activity of amidopropyl dimethylamines (MAPD, OAPD and EAPD) and their amidopropyl trimethyl quaternary ammonium versions (MAPT, OAPT and EAPT) against *A. castellanii* (ATCC 50370) cysts over a 24 h period at a concentration of 0.025 mM. Error bars show the standard error of the mean (SEM) from triplicate experiments.

Figure 1 shows that at a concentration of 0.025 mM, all APDs exhibited a time-dependent reduction in *A. castellanii* cyst viability over 24 h, with MAPD, OAPD, EAPD, MAPT, OAPT and EAPT achieving a log_10_ reduction of 1.67, 2.58, 2.42, 2.58, 3.25 and 2.92, respectively.

**Figure 2:**
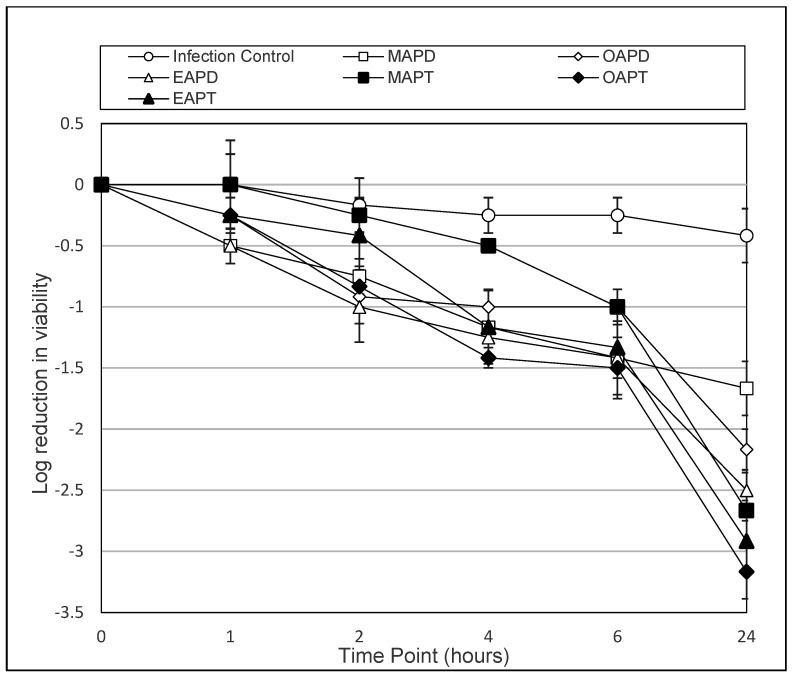
The above graph shows the antimicrobial activity of amidopropyl dimethylamines (MAPD, OAPD and EAPD) and their amidopropyl trimethyl quaternary ammonium versions (MAPT, OAPT and EAPT) against *A. polyphaga* (ATCC 30461) cysts over a 24 h period at a concentration of 0.025 mM. Error bars show the standard error of the mean (SEM) from triplicate experiments.

Figure 2 shows that at a concentration of 0.025 mM, all APDs exhibited a time-dependent reduction in *A. polyphaga* cyst viability over 24 h, with MAPD, OAPD, EAPD, MAPT, OAPT and EAPT achieving a log_10_ reduction of 1.67, 2.17, 2.50, 2.67, 3.17 and 2.92, respectively.

## 4. Discussion

Microbial keratitis is a potentially sight-threatening infection frequently associated with contact lens wear, caused by a range of pathogens, including bacteria, fungi, and free-living amoebae. Weekly and monthly contact lens users are advised to disinfect their lenses daily, but several risk factors, such as poor lens hygiene and the selection of multipurpose solutions (MPSs), increase the likelihood of infection. Acanthamoeba keratitis (AK), caused by *Acanthamoeba* species, is of particular concern due to the organism’s ability to form highly resistant cysts that are difficult to eradicate with standard disinfection methods. When AK develops, treatment is prolonged and complex, often requiring several months to achieve resolution. As a result, there is a crucial need to identify alternative agents for incorporation into MPS for lens disinfection and for AK management.

This study evaluated novel amidopropyl dimethylamines (APDs) and amidopropyl quaternary trimethylammoniums (APTs) for their activity against cysts of *A. castellanii* and *A. polyphaga*. The compounds were first screened to determine the minimum effective concentrations against the organisms, followed by an assessment of their inactivation kinetics against these microbes over a 24 h period.

### 4.1. Minimum Trophozoite Amoebicidal Concentration (MTAC) and Minimum Cysticidal Concentration (MCC)

Compared to MAPD (C14), several compounds exhibited varying activities based on their MTAC results. PAPD (C16) displayed amoebicidal activity at 0.023 mM, which was slightly higher compared to MAPD, whereas LiAPD (C18:3), EAPD (C18:1 *trans*) and OAPD (C18:1 *cis*) each had higher activity, with each having identical MTAC values of 0.011 mM. These unsaturated dimethylamines with an alkyl chain length of C18 were able to demonstrate enhanced activity against *Acanthamoeba* trophozoites. In particular, LiAPD demonstrated the greatest activity at 0.005 mM compared to 0.025 mM with MAPD.

Conversely, compounds that exhibited considerably reduced activity compared to MAPD with significantly higher MTAC values include LAPD (18:2) at 0.171 mM, DAPD (C10) at 0.244 mM and DoDAPD (C12) at 0.055 mM. Unlike the unsaturated LiAPD, EAPD and OAPD, saturated SAPD (C18) demonstrated reduced activity compared to MAPD, with an MTAC at 0.678 mM compared to 0.025 mM with MAPD.

The minimum cysticidal concentration (MCC) represents the lowest concentration required to achieve the complete killing of *Acanthamoeba* cysts, the most resistant form of the *Acanthamoeba*. For MAPD, which is used in a number of commercially available contact lens solutions, the MCC value was 0.050 mM for *A. castellanii* and 0.1 mM against *A. polyphaga*. LiAPD (18:3) showed greater activity, with an MCC value of 0.086 mM. However, OAPD (18:1) and its *trans* isomer EAPD (18:1) exhibited MCC values of 0.085 mM, displaying better activity than MAPD with *A. polyphaga* cysts but slightly lower activity against *A. castellanii* cysts. In contrast, compounds with higher MCC values were indicative of lower potency against *Acanthamoeba* cysts, which included DoDAPD (C12), DAPD (C10) at 0.975 mM, PAPD (C16) at 0.367 mM and LAPD (18:2) at 0.686 mM. Compared to the saturated dimethylamine SAPD (C18) with the highest MCC of 1.356 mM, the unsaturated EAPD, OAPD and LiAPD emerged as the most promising candidates, exhibiting slightly better or comparable potency to MAPD.

Among the compounds tested, the trimethylammoniums displayed significant cysticidal activity against *Acanthamoeba* cysts, apart from MAPT. OAPT (C18:1 *cis*) and EAPT (C18:1 *trans*) displayed significantly greater activity when compared to their dimethylamine counterparts, both with an MCC value of 0.010 mM compared to 0.025 mM for MAPD for both strains. In contrast, MAPT exhibited the lowest efficacy among the synthesised trimethylammoniums, with MCC values of 0.191 mM against *A castellanii* and 0.382 mM against *A. polyphaga* cysts.

### 4.2. Time–Kill Assays

Table 3, Table 4, Table 5 and Table 6 and Figure 1 and Figure 2 demonstrate the time-dependant kinetic activity of 0.025 mM of the selected APDs and APT compounds against *Acanthamoeba* cysts over a 24 h period. As demonstrated, all compounds showed a decrease in the viability of cysts compared to the control (*p* < 0.004) after 24 h. From the 12 compounds evaluated against the cysts of both species, the most active compounds were found to be MAPD, OAPD, EAPD, MAPT, OAPT and EAPT. As demonstrated in Figure 1 and Figure 2, all compounds demonstrated superior activity to MAPD at the 24 h time point.

With A. *castellanii,* MAPD achieved a log_10_ reduction of 1.67 compared to OAPD, EAPD, MAPT, OAPT and EAPT achieving a log_10_ reduction of, 2.58, 2.50, 2.42, 3.25 and 2.92, respectively (Figure 1). The results with EAPD were found to be not significant (*p* > 0.05), whereas OAPD (*p* = 0.0246), MAPT (*p* = 0.0246), OAPT (*p* = 0.0019) and EAPT (*p* = 0.0002) were found to have significantly enhanced activity.

With A. polyphaga, MAPD achieved a log_10_ reduction of 1.67 compared to OAPD, EAPD, MAPT, OAPT and EAPT achieving a log_10_ reduction of, 2.17, 2.50, 2.67, 3.17 and 2.92, respectively (Figure 2). The results with OAPD and EAPD were found to be not significant (*p* > 0.05), whereas MAPT (*p* = 0.0272), OAPT (*p* = 0.0049) and EAPT (*p* = 0.0009) were found to have significantly enhanced activity.

OAPT was the most potent APT among both APDs and APTs displaying significant activity, with a 3.17 log_10_ reduction and 3.25 log_10_ reduction in both strains *A. castellanii* and *A. polyphaga*, respectively, after 24 h (*p* < 0.0001). This is the first time that these compounds have been demonstrated to have activity against the trophozoites and cysts of *Acanthamoeba* spp., with the exception of MAPD (C14), which has previously been reported to produce a 3-log reduction in *Acanthamoeba* cyst numbers in 6 h at a concentration of 0.032 [15].

### 4.3. Mechanism of Action

The exact mechanism of action of these compounds is unknown but it is most likely that these surfactants have a detergent like action which disrupts with the close packing structure of the components of the *Acanthamoeba* lipid bilayer causing the failure of membrane integrity [23]. The first step in the antimicrobial activity involves the positively charged polar head group of the APDs and APTs interacting with the negatively charged polar head groups of the membrane phospholipids of the *Acanthamoeba* plasma membrane [24].

The results from this study suggest that the compounds with amidopropyl-dimethylamine and quaternary ammonium head groups interact avidly with *Acanthamoeba* membrane phospholipids in trophozoites and cysts as demonstrated by their amoebicidal (MTAC) and cysticidal (MCC) activity. The binding of the head group is believed to be followed by the insertion of the long fatty acid carbon chain of the molecule into the bilayer. Then, depending on the length of the chain this can cause a mismatch in chain length between the carbon chains in the surfactant and those in the membrane bilayer [24].

This in turns leads to the creation of free volume below the surfactant molecule in the hydrophobic region of the bilayers, leading to the perturbation of the membrane and decreasing its stability [24]. Then, as more and more surfactant molecules interact, by slotting into the membrane, the level of perturbation increases, eventually leading to the complete breakdown of membrane integrity [24]. The results from this study indicate that the optimal chain length for antimicrobial activity with the amidoamines is in the C12–C14 range for the saturated chains as reduced activity is observed with the shorter C10 and longer C16 and C18 straight-chain-length compounds. This observation is supported by the finding from a similar study against bacteria which determined that C12 and C15 compounds were optimal for antimicrobial activity when tested against *E. coli* and *S. aureus,* respectively [25].

The reduced activity of the shorter-chained C10 compound in this study may be explained by this theory for the mechanism of activity. It is believed that the shorter-chained molecules penetrate only a short distance into the lipid bilayer and are therefore less able to disrupt membrane integrity. Moreover, the absence of activity with the longer-chained compound C18 may be explained by the chemical properties of the surfactants. It is known that as the carbon chain length of the surfactant increases, the tendency for the surfactant molecule to form a micelle increases, thus decreasing the amount of the monomeric surfactant available to interact with the lipid membrane [26].

Furthermore, this study showed that the presence of unsaturated bonds in the carbon chain influenced activity as the monosaturated C18:1 and polyunsaturated C18:2/C18:3 compounds all showed significantly greater activity compared to the saturated C18 straight-chain compound (SAPD). This is likely to be caused by the greater perturbation of the *Acanthamoeba* membrane phospholipids by the unsaturated test compounds due to the bent conformation of the fatty acid chain.

## 5. Conclusions

In conclusion, this study highlighted a series of amine-containing surfactants that have superior activity compared to MAPD against the trophozoites and cysts of *Acanthamoeba.* These compounds warrant further study for their potential as treatment for AK and for their incorporation in contact lens disinfectants. The activity of these compounds is variable but is dependent on the type and charge of the polar head group and also on the length of the carbon chain.

## Figures and Tables

**Table 1:** The table shows the minimum trophozoite amoebicidal concentration (MTAC) and minimum cysticidal concentration (MCC) of the synthesised tertiary amidoamines against *A. castellanii* (ATCC 50370) and *A. polyphaga* (ATCC 30461) and toxicity assays performed against mammalian epithelial cells (MCT). Minimum cytotoxic concentration (MTC) against human epithelial cell line (Hep2).

Compound	Abb.	Alkyl Chain Length	mM				
			*A. castellanii* ATCC 50370		*A. polyphaga* ATCC 304610		*Hep 2*
			MTAC	MCC	MTAC	MCC	MCT
Capramidopropyl dimethylamine	CAPD	(C10)	0.244	0.975	0.244	0.975	0.061
Lauramidopropyl dimethylamine	DoDAPD	(C12)	0.055	0.110	0.055	0.220	0.110
**Myristamidopropyl** **dimethylamine**	**MAPD**	**(C14)**	**0.025**	**0.050**	**0.025**	**0.100**	**0.200**
Palmitamidopropyl dimethylamine	PAPD	(C16)	0.023	0.367	0.011	0.367	0.046
Stearamidopropyl dimethylamine	SAPD	(C18)	0.678	1.356	1.356	1.356	0.011
Linoleamidopropyl dimethylamine	LAPD	(C18:2)	0.171	0.686	0.171	0.686	0.343
Linolenamidopropyl dimethylamine	LiAPD	(C18:3)	0.011	0.086	0.005	0.086	0.086
Elaidamidopropyl dimethylamine	EAPD	(C18:1) (*trans*)	0.011	0.085	0.011	0.085	0.021
Oleamidopropyl dimethylamine	OAPD	(C18:1) (*cis*)	0.011	0.085	0.043	0.085	0.021

**Table 2:** The table below shows the minimum cysticidal concentration (MCC) and the minimum trophozoite amoebicidal concentration (MTAC) of the synthesised quaternary (QUAT) trimethylammoniums (MAPT, EAPT, and OAPT) required against *A. castellanii* (ATCC 50370) and *A. polyphaga* (ATCC 30461) and the toxicity assay performed against mammalian epithelial cells. Minimum cytotoxic concentration (MTC) against human epithelial cell line (Hep2).

Compound	Abb.	Alkyl Chain Length	mM				
			ATCC 50370		ATCC 30461		*Hep 2*
			MTAC	MCC	MTAC	MCC	MCT
Myristamidopropyl trimethylammonium	MAPT	C14 QUAT	0.095	0.191	0.191	0.382	0.048
Elaidamidopropyl trimethylammonium	EAPT	C18:1 *trans* QUAT	0.005	0.020	0.005	0.020	0.041
Oleamidopropyl trimethylammonium	OAPT	C18:1 *cis* QUAT	0.003	0.010	0.003	0.005	0.041

**Table 3:** Efficacy of APDs against *Acanthamoeba castellanii* (ATCC 50370) over a 24 h period.

A. castellanii (ATCC 50370)						
Amidopropyl Dimethylamines (APDs) (0.025 mM)						
Test Compound	Average Log_10_ Reduction in Viability with Exposure (Hours)					
	0	1	2	4	6	24
PAPD	0.00	0.08	0.08	0.08	0.17	0.58
CAPD	0.00	0.08	0.08	0.25	0.42	0.50
LAPD	0.00	0.00	0.08	0.08	0.58	0.75
SAPD	0.00	0.08	0.00	0.08	0.42	0.67
LaAPD	0.00	0.17	0.33	0.42	0.67	1.33
LnAPD	0.00	0.17	0.25	0.25	0.58	1.42
MAPD	0.00	0.50	0.75	1.17	1.58	1.67
OAPD	0.00	0.42	1.00	1.08	1.17	2.58
EAPD	0.00	0.50	1.08	1.25	1.33	2.42

**Table 4:** Efficacy of APDs against *Acanthamoeba polyphaga* (ATCC 30461) over a 24 h period.

A. polyphaga (ATCC 30461)						
Amidopropyl Dimethylamines (APDs) (0.025 mM)						
Test Compound	Average Log_10_ Reduction in Viability with Exposure (Hours)					
	0	1	2	4	6	24
PAPD	0.00	0.25	0.42	0.50	0.75	1.33
CAPD	0.00	0.00	0.25	0.42	0.58	0.58
LAPD	0.00	0.08	0.00	0.25	0.50	1.67
SAPD	0.00	0.08	0.08	0.08	0.42	1.00
LaAPD	0.00	0.08	0.00	0.08	0.17	1.17
LnAPD	0.00	0.17	0.17	0.25	0.33	0.92
MAPD	0.00	0.50	0.75	1.17	1.42	1.67
OAPD	0.00	0.25	0.92	1.00	1.00	2.17
EAPD	0.00	0.50	1.00	1.25	1.42	2.50

**Table 5:** Efficacy of APTs against *Acanthamoeba castellanii* (ATCC 50370) over a 24 h period.

A. castellanii (ATCC 50370)						
Amidopropyl Trimethylammoniums (APTs) (0.025 mM)						
Test Compound	Average Log_10_ Reduction in Viability with Exposure (Hours)					
	0	1	2	4	6	24
MAPT	0.00	0.17	0.33	0.67	1.08	2.58
OAPT	0.00	0.33	1.00	1.33	1.58	3.25
EAPT	0.00	0.33	0.50	1.25	1.42	2.92

**Table 6:** Efficacy of APTs against *Acanthamoeba polyphaga* (ATCC 30461) over a 24 h period.

A. polyphaga (ATCC 30461)						
Amidopropyl Trimethylammoniums (APTs) (0.025 mM)						
Test Compound	Average Log_10_ Reduction in Viability with Exposure (Hours)					
	0	1	2	4	6	24
MAPT	0.00	0.00	0.25	0.50	1.00	2.67
OAPT	0.00	0.25	0.83	1.42	1.50	3.17
EAPT	0.00	0.25	0.42	1.17	1.33	2.92

## Data Availability

The original contributions presented in this study are included in the article. Further inquiries can be directed to the corresponding authors.
